# Addressing Uncertainty in Informal Familial Caregivers of Stroke Survivors: A Systematic Meta-Ethnography

**DOI:** 10.3390/ijerph191711116

**Published:** 2022-09-05

**Authors:** Gabriella T. Ponzini, Brenna Kirk, Sarah E. Segear, Elizabeth A. Claydon, Elizabeth B. Engler-Chiurazzi, Shari A. Steinman

**Affiliations:** 1 Department of Psychology, West Virginia University, Morgantown, WV 26506, USA; 2 School of Public Health, West Virginia University, Morgantown, WV 26506, USA; 3 Department of Neurosurgery, Tulane University, New Orleans, LA 70118, USA

**Keywords:** uncertainty, coping, stroke, caregiver, meta-ethnography

## Abstract

**Background:** Informal familial caregivers of stroke survivors experience uncertainty that begins at the time of the stroke event and continues into home-based care. The uncertainty faced by caregivers contributes to poor mental and physical health outcomes. **Objective:** This review details the factors associated with, impacts of, and coping skills used to manage uncertainty across the caregiving trajectory. By defining uncertainty reduction and tolerance recommendations, this review also builds upon the *Stroke Caregiver Readiness Model* to improve preparedness following the stroke event. **Methods:** A meta-ethnographic review was systematically conducted on thirteen qualitative studies with 218 participants from four countries. The Critical Appraisal Skills Programme (CASP) was used to assess study quality. **Results:** Following the stroke event, caregivers reported a lack of knowing (e.g., about the cause of the stroke event and survivor prognoses) as contributing to post-stroke uncertainty. As a result of this uncertainty, caregivers expressed concerns about their abilities to navigate caregiving responsibilities and how to plan for the future. Longer-term concerns (e.g., managing finances) and feelings of hopelessness occurred after discharge. Still, caregivers identified strategies to manage uncertainty. Caregiver coping skills included present-focused thinking, gratitude, faith, humor, and social support. **Conclusions:** The uncertainty faced by informal familial caregivers of stroke survivors is pervasive and changes across time. Uncertainty reduction and tolerance interventions can be used to build upon caregiver strengths and promote preparedness across the caregiving trajectory.

## 1. Introduction

In the United States alone, more than 795,000 individuals have a stroke each year [1]. Although many individuals now survive a stroke event, strokes represent the leading cause of adult disability [1]. The physical, socioemotional, and functional effects of a stroke are associated with increased personal and public costs [2]. As such, many stroke survivors require at-home caregiving [3], and family members of stroke survivors are often transitioned into caregiving roles. Of the approximately 4.8 million stroke survivors, there are roughly 3.5 million unpaid (i.e., informal) familial caregivers [4].

On average, stroke survivors spend approximately 4.7 days in the hospital following a stroke event [5]. After hospitalization, there are several types of post-acute care options available depending on the rehabilitative needs of the survivor. Inpatient rehabilitation facilities represent a hospital-environment option with continuous care. Such facilities account for approximately 24% of post-acute patients and the typical stay is about one week [6]. Alternatively, skilled nursing facilities, which provide post-acute care to about 30.8% of survivors, allow for a less intensive option that focuses on physical, occupational, and speech rehabilitation. For patients with Medicare, about 45% of individuals are released home immediately after their hospitalization. Home-based rehabilitative care may be available for these stroke survivors; however, options are typically short-term [6]. Regardless of the post-acute care option, informal familial caregivers typically begin providing at-home caregiving within weeks of the stroke event.

The transitory periods from hospital to home-based care have been considered crisis points for stroke survivors and their families [7]. At first, caregivers must face the reality of the stroke event, the unpredictability of survivor prognoses, and plan for rehabilitation services. Then, caregivers must confront the reality that full recovery (e.g., cognitive and functional impairments improved to pre-stroke status) may not be possible for many survivors. Approximately 32–44% of stroke survivors require assistance with activities of daily living three to six months post-stroke [8], and familial caregivers report feeling underprepared to meet patients’ basic needs [9]. Once at home, caregivers face the long-term uncertainties associated with the stroke event: worries related to finances, future-plans, and the stability of the survivors and their own health status [7]. As such, informal familial caregivers of stroke survivors must manage both the immediacy of the stroke event and the highly variable nature of patient recovery [10].

The uncertainty endured by informal caregivers of stroke survivors is not without its consequences. Roughly half of post-stroke caregivers experience symptoms of depression and anxiety [11,12,13], for which uncertainty is a transdiagnostic risk factor. Further, heightened levels of distress increase their risks of mortality, strokes, and coronary heart disease [14,15]. As the health of caregivers deteriorates, so does their ability to provide effective care to the stroke survivor [7]. In short, understanding and addressing the uncertainty faced by informal familial caregivers of stroke survivors is necessary to promote the wellbeing of those affected by a stroke event.

To enhance caregiver preparedness, Lutz and colleagues (2016) [16] proposed the *Stroke Caregiver Readiness* Model. This model first suggests a needs-based assessment for the caregiver and stroke survivor, as well as an evaluation of the caregiver’s commitment and capacity to provide care [16]. Then, individualized identification of resources, skills training, home preparation, as well as self-care strategies for the caregiver can be provided. While this framework does not focus on uncertainty as a concept, caregiver uncertainty may undermine readiness through the introduction of doubt and distress. Moreover, understanding caregiver uncertainty as it relates to readiness can provide a deeper understanding of how to best assist caregivers as they transition into their new roles. Accordingly, through a systematic synthesis of previous research, we analyze the factors associated with, impacts of, and coping skills to manage uncertainty for caregivers during the post-stroke crises (i.e., the stroke event and discharge). Conclusions inform intervention recommendations that can further increase caregiver preparedness via uncertainty reduction and management strategies.

## 2. Methods

### 2.1. Design

A meta-ethnographic approach was used to synthesize the findings of qualitative studies [17]. This approach allows for a systematic, re-interpretation of conceptual data (e.g., themes) across studies to draw conclusions and identify directions for future research [17]. Meta-ethnographies involve the development of higher-order themes (i.e., lines of argument) from a comparative synthesis of participant (i.e., first order) and author (i.e., second order) quotes across qualitative research studies (see Sattar et al., 2021 for greater detail) [18]. Meta-ethnographic syntheses are rigorous and the most utilized approach in health-related research [19]. To ensure systematic reporting of the available data, the enhancing transparency in reporting the synthesis of qualitative research (ENTREQ) checklist [20] and the eMERGe meta-ethnography reporting guidelines [21] were also utilized (see below for details).

### 2.2. Criteria for Including Studies

Included studies needed to be empirical, qualitative, and published in English in a peer-reviewed journal. All studies must have recruited a sample of informal (i.e., unpaid) familial caregivers of stroke survivors. Further, studies needed to evaluate or draw conclusions about factors associated with, impacts of, and coping skills to manage uncertainty. Studies were not limited by country of origin. All included studies were published prior to March of 2021.

### 2.3. Systematic Search Strategy

A systematic literature search was conducted by S.E.S. and reviewed by G.T.P. This search aimed to be purposeful (e.g., concept saturation) rather than inclusive of all related research, as consistent with meta-ethnography guidelines [16]. To identify key words and abstracts, the following search terms were utilized: “stroke” OR “post-stroke”, AND “caregiver” OR “caretaker” OR “carer”, AND “uncertainty” OR “anxiety”. Searches were conducted using CINAHL, PubMed, PsycINFO, and Google Scholar.

### 2.4. Identification of Studies

Title and abstracts were screened by S.E.S. Full-text reviews were carried out by G.T.P. and S.E.S. to determine their appropriateness for inclusion. There were no discrepancies regarding the included studies.

### 2.5. Data Extraction

Each study was reviewed independently by G.T.P., B.K., and S.E.S. The following data were extracted from each article by S.E.S.: author names, year of publication, country of data collection, sample size, caregiver relation (e.g., spouse), age, gender, and race of caregivers and stroke survivors. Then, G.T.P. extracted study aims, qualitative methods, sampling methods, data collection methods, lengths of interviews, data analysis methods, and main findings.

### 2.6. Quality Appraisal

The included studies were subjected to the Critical Appraisal Skills Programme (CASP) checklist for Qualitative Studies (CASP) [22]. The CASP checklist includes 10 items that assess research clarity, appropriateness of qualitative methods, research design, recruitment strategy, data collection methods, researcher bias, ethical considerations, rigor of data analysis, reporting of findings, and overall value of the research.

### 2.7. Analysis and Synthesis

The analysis was conducted following established meta-ethnographic guidelines [16]. G.T.P. extracted first-order (participant quotes) and second-order (author interpretations of participant data) constructs, which were taken from studies verbatim to be utilized for analysis and synthesis. Then, G.T.P. grouped studies according to factors associated with, impacts of, and coping skills identified to manage uncertainty by caregivers. Next, G.T.P. and B.K. conducted independent comparisons across studies to develop a list of related themes. Themes were agreed upon and clustered based on shared concepts to create third-order constructs (i.e., the reviewers’ higher order interpretations based on analysis). Lastly, G.T.P. and B.K. independently re-coded studies as refuting, dissimilar but not disproving the line of argument, or reciprocation of the third-order constructs.

### 2.8. Positionality Statements

To promote reflexivity and transparency with regards to data interpretation, the authors have self-identified their areas of expertise, relationship to stroke survivorship and/or caregiving, and their demographic identities.

G.T.P. is a PhD Candidate in clinical psychology with expertise in the research and treatment of anxiety disorders and obsessive-compulsive disorder. She was a predoctoral fellow on the ‘Stroke and Its Comorbidities’ NIH T32 grant at West Virginia University. During her time as a fellow, she evaluated the role of post-stroke anxiety on the well-being of long-term survivors and their families. She is quantitatively trained but has spent the last two years receiving mentorship on qualitative methods. Although she has never been a caregiver, she has had a close family member survive a stroke. B.K. is a PhD Candidate with over seven years of experience in qualitative health research. She does not have research expertise related to stroke but has acted as an informal caregiver for relatives who have experienced stroke, dementia, and physical disability related to limb amputation. S.E.S. is Psychometrist for an outpatient neuropsychology department. She does not have research expertise related to stroke but has had experience assessing stroke patients and caregivers. E.A.C is an assistant professor in public health with six years of qualitative research experience. Her research is primarily focused on mental health and public health interventions. She does not have research expertise related to stroke but has had family members act as informal caregivers of relatives. E.B.E.-C. is a behavioral neuroscientist with expertise in preclinical models of stroke and its comorbidities. She has a close family member who suffered from a stroke. S.A.S. is a clinical psychologist that specializes in anxiety and obsessive-compulsive disorder and has published on the intolerance of uncertainty. Although she has some qualitative research experience, she typically uses quantitative methodology. All authors identify as white, heterosexual, cis-gender females.

## 3. Results

### 3.1. Searches

**Figure 1:**
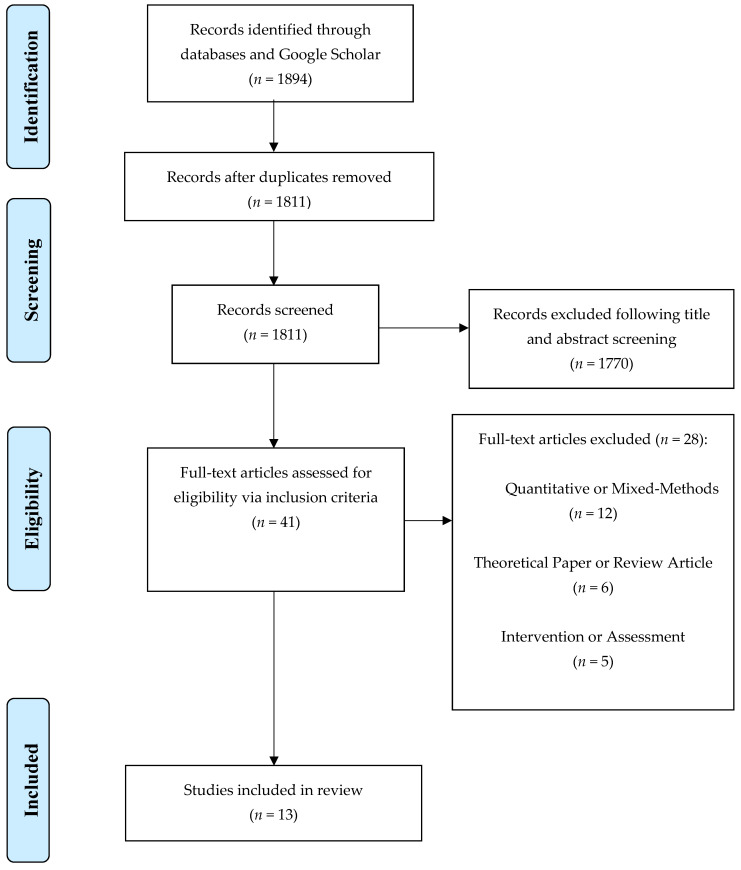
Preferred Reporting Items for Systematic Reviews and Meta-analyses (PRISMA) flow diagram [23].

A total of 1894 articles were identified from the initial searches. Duplicates across searches (*n* = 83) were removed. The remaining 1770 articles were screened for relevant titles and abstracts. Of these articles, 41 were deemed appropriate for full-text reads. Following article review, 13 studies remained for the present review. See Figure 1 for details.

### 3.2. Study Characteristics

Table 1 shows demographic characteristics and Table 2 shows methodological characteristics for the included studies.

### 3.3. Quality Appraisal (CASP)

For each study, G.T.P. and B.K. independently coded each item of the checklist as YES (i.e., manuscript allows for a conclusion that criteria are met), NO (i.e., manuscript allows for a conclusion that criteria are *not* met), or UNSURE (i.e., manuscript does not allow for a conclusion to be drawn). Then, G.T.P. and B.K. reviewed coding. Agreement between coders prior to review was 90% and was 100% following discussions. Across the included studies, quality was strong. All studies provided enough information to draw conclusions on *at least* seven of the ten CASP items. Studies most often did not provide enough detail to determine the justification of their research design or address research bias and the relationship between researchers and participants. Still, the overall quality of the studies allowed for all to be included and weighed equally in the present review. See Table 3 for agreed upon quality appraisal ratings.

### 3.4. Meta-Ethnography Analysis

Table 4 shows the key concepts derived from constant comparison. Provided the significant overlap across study findings, a subset of representative quotes (chosen for their descriptiveness) is used to demonstrate constructs.

### 3.5. Lines of Argument

#### 3.5.1. Factors Associated with Uncertainty

Across studies, the unexpected nature of the stroke event served as a catalyst for caregiver uncertainty. Following the event, caregivers detailed their lack of knowledge as maintaining their uncertainty. Specifically, they reported an inability to “know” the cause of the event [26,30,34], the stroke survivors’ prognosis [25,27,29,31,32,33,34,35], the safety of the survivor if left alone [24,27,29,30,36], the support options available [27,29,35,36], and how to provide care to the survivor [27,30,34,35,36]. While only expressed by some caregivers in one study [35], the loss of intimacy with and lack of empathy from survivors also contributed to uncertainty.

Of the factors associated with uncertainty, none were associated with caregiver experience (i.e., prior caregiving experience or novice caregivers). Still, uncertainty about the cause of the stroke event and survivor prognoses persisted from acute settings to post-discharge. Longer-term worries (e.g., concerns about the safety of the survivor), however, were only expressed post-discharge.

#### 3.5.2. Impacts of Uncertainty

As a result of this uncertainty, caregivers reported worries about their abilities to successfully navigate their newfound responsibilities. Specifically, they noted concerns about their abilities to manage finances [24,35,36], effectively plan for the future [24,26,29], identify others who would assume caregiving responsibilities for the survivor if they themselves fell ill [25,27,35], and ways to cope with the stroke event [26,35]. The lack of clarity surrounding these concerns resulted in feelings of hopelessness [24,25,27].

Of the impacts of uncertainty, none were associated with caregiver experience. Concerns about caregivers’ abilities to plan for the future were noted specifically in caregivers whose stroke survivors were in rehabilitative settings. Additionally, worries associated with finances, caring for the survivor if they themselves fell ill, and feelings of hopelessness were specific to caregivers providing care-post-discharge. Across both acute and home-based settings, caregivers reported concerns about how to cope with the stroke event, pointing to the longstanding effects of uncertainty.

#### 3.5.3. Coping Strategies to Manage Uncertainty

Encouragingly, caregivers were able to identify effective coping mechanisms to manage their distress. Specifically, caregivers engaged in present-focused thinking [24,29,31,32,33,34,36], acceptance [32,36], gratitude and positivity [29,31,34,35], and faith (both spiritual and hope-based); [30,31,32].

Caregivers also took an active role in the stroke survivors’ recovery through increasing self-reliance [28,29,32], seeking information [28,29,36], establishing routines [29,32,36], and engaging in watchful monitoring of the stroke survivor [32,36]. Some studies found that caregivers who felt it was their duty to provide care embraced caregiving more readily [32,35].

While several caregivers reported using humor to cope with distress [29,31,34], they also found comfort in reliving memories from the past [32] and continuing with prior hobbies [31]. Further, while most caregivers reported social support as a coping skill [26,29,31,32], a few caregivers noted the need to “turn inward”, and minimize outside contact [32]. Although caregiver experience was not found to be associated with any notable benefits across studies, one study found that experienced caregivers expressed less uncertainty and greater confidence in their abilities to cope with the stroke event compared with newer caregivers [29].

Lastly, effective coping skills were identified by caregivers across the caregiving trajectory (acute hospitalization through post-discharge). Across all stages of post-stroke caregiving, present-focused thinking, gratitude, faith, humor, and social support were noted as effective coping strategies by caregivers. For caregivers whose stroke survivors were still in acute care, continuation of prior hobbies was identified as especially important. Some strategies, however, appeared to be more useful following discharge. That is, acceptance, increased self-reliance, information seeking, routine establishment, watchful monitoring of the stroke survivor, embracing their duty, and reliving memories were all specific to caregivers providing home-based care.

## 4. Discussion

The present meta-ethnographic review details factors related to, impacts of, and coping strategies to manage uncertainty in informal familial caregivers of stroke survivors. For caregivers, the stroke event served as the catalyst for uncertainty. Then, in accordance with the stroke crises [16], caregivers reported concerns about coping with the stroke event and survivors’ abilities to recover while in inpatient settings. Concerns about how to cope with the stroke event also persisted into the delivery of home-based care. Our findings suggest that caregiver concerns about the safety of the survivor, available support, and abilities to provide care, as well as worries about finances, caring for the survivor if they themselves fell ill, and feelings of hopelessness were specific to providing care post-discharge. Of note, as caregivers progress from the crisis of the stroke event to the crisis of discharge, their focus shifted more towards attending to the needs of survivors and away from caring for themselves [7]. Encouragingly, however, caregivers were able to identify coping strategies they perceived as helpful to minimize the negative effects of uncertainty.

In all, the course of uncertainty experienced by informal familial caregivers appears to parallel the path to recovery for stroke survivors: both trajectories are influenced by various and sometimes unpredictable factors which change across time. This long-term unpredictability and the constantly changing nature of caregiving may explain why prior caregiving experience was not associated with uncertainty reduction or improved uncertainty tolerance across studies. Moreover, although factors contributing to uncertainty appeared to change across time (e.g., concerns about *how* to provide care for the stroke survivor to concerns about *who* would care for the stroke survivor if the caregiver fell ill), the experience of uncertainty itself remained stable throughout the caregiving trajectory. Thus, in addition to supporting the changing needs of stroke survivors, there is also a need to improve the perceived preparedness of informal familial caregivers to reduce the associated uncertainty and distress of caregiving.

In the present review, we provide a strong foundation for understanding informal familial caregiver uncertainty as it relates to stroke survivors. The quality of the included studies (as evidenced by the CASP review) and the rigor with which the review was conducted (use of eMERGe guidelines and ENTREQ checklist) enhance the trustworthiness of the abovementioned conclusions. Additionally, study results were largely parsimonious, suggesting a shared experience of uncertainty across countries of origin, levels of post-acute care, and experiences of caregiving during the decades which the included research was conducted. Accordingly, to assist caregivers in the management of their uncertainty across the caregiving trajectory, we propose the use of interventions grounded in research that build upon caregiver strengths and can be implemented shortly following a stroke event. Such interventions also expand on recommendations by Lutz and colleagues’ *Stroke Caregiver Readiness Model* (2016; e.g., targeted skills-based trainings, as well as family counseling) to further support caregiver readiness through uncertainty reduction and tolerance.

### 4.1. Implications for Practice: Uncertainty Reduction and Tolerance

#### 4.1.1. Uncertainty Reduction: Education and Skills-Based Peer Support Groups

Educational and skills-based interventions have aimed at improving caregiver knowledge and perceived competence by providing information on stroke events, practical skills for caregiving, and general coping skills (e.g., emotion regulation) [37]. These interventions, which are often delivered by nurses, social workers, or psychologists, have demonstrated beneficial effects on stroke caregivers’ mental health [37,38,39,40,41]. While caregivers in the current review reported a desire to “know” (e.g., about the stroke event, caregiving skills, and survivor prognoses), they also identified concerns about the accessibility of information provided to them by hospital staff. To address this concern and better support the needs of the caregivers, we suggest the addition of education and skills-focused peer-support groups. Peer-support groups permit those with similar experiences to provide practical and emotional support during the management of health conditions [42]. Peer support groups for caregivers of stroke survivors have led to an increased sense of community and awareness, as well as perceived empowerment for caregivers [43,44]. Accordingly, trained peer-support specialists (i.e., group leaders with personal experience) [45] may also be able to increase the accessibility of educational materials and skills-based training. By delivering such interventions across the caregiving trajectory (e.g., in-person groups during acute care and video groups during home-based care), these interventions may help to reduce the negative impact of prognostic expectations regarding the stroke survivor’s recovery. That is, by providing accessible information via a community-driven format, caregivers may be able to better prepare for the longer-term outcomes associated with unpredictable course of this illness.

#### 4.1.2. Uncertainty Tolerance: Cognitive Behavioral Strategies

Caregivers in the current review identified numerous strategies to cope with the uncertainty brought about by the stroke event. In addition to these strategies, psychotherapeutic interventions such as cognitive behavioral therapy (CBT) have been effective in reducing symptoms of anxiety and depression in caregivers of stroke survivors [46]. Specifically, skills such as cognitive restructuring (i.e., modifying existing beliefs to be more realistic and helpful) can help challenge caregivers’ beliefs about their ability to tolerate uncertainty (e.g., “If I don’t know their prognosis, I won’t be able to manage my distress”) or their interpretation of uncertain events as negative (e.g., “If I don’t know their prognosis, then it must be poor”). Likewise, cognitive restructuring can be used to formulate more neutral or positive beliefs about caregiver preparedness during transitory periods of care (e.g., “Although I have not had to be a caretaker before, I have skills that will allow me to be successful in this role”). In addition, behavioral strategies based in acceptance and commitment therapy (ACT), such as values-identification and goal setting, may help caregivers feel greater control over their caregiving experience [47]. Given that caregivers identified ACT-based skills (i.e., present-focused thinking and engaging in meaningful activities) as strategies to cope with uncertainty, additional acceptance-based skills may enhance caregivers’ abilities to shift their focus from what they cannot control to what they can control [7,16]. By attending individual psychotherapy or incorporating CBT and ACT-based skills into the abovementioned peer support groups, caregivers may experience enhanced readiness and preparedness in navigating their caregiving trajectories.

### 4.2. Limitations of the Review

While this review had numerous strengths, limitations must also be considered. First, within the studies reviewed, researcher bias was poorly assessed and addressed by study authors. Accordingly, the interpretation of participants’ data may have been influenced by study authors’ beliefs. Further, the authors of this review want to acknowledge their own potential bias (including personal experiences of informal familial caregiving) on their interpretation and synthesis of the data. Moreover, the search strategy utilized was purposeful and aimed to reach concept saturation as it related to informal familial caregiver uncertainty and related coping strategies to manage uncertainty. We acknowledge, however, that studies on relevant topics (e.g., caregiver burden) may have been missed due to the specificity of our search terms. Lastly, the authors want to acknowledge that the recommendations put forth in this paper may not be inherently accessible to all informal familial caregivers of stroke survivors. Historically, individuals who have been marginalized within the healthcare system (e.g., racial/ethnic minorities, low-income individuals, and those in LGBTQIA+ communities) have been burdened with limited access to resources and poorer quality care. Furthermore, given that the samples in the studies reviewed were largely white, the abovementioned findings about uncertainty as well as the intervention recommendations may not be generalizable. It is our strong recommendation that any future interventions related to caregiver uncertainty are developed with, evaluated by, and targeted to address the specific needs of marginalized communities.

### 4.3. Conclusions

This review provides a detailed account of uncertainty experienced by informal familial caregivers of stroke survivors to demonstrate how such uncertainty undermines readiness and preparedness across the caregiving trajectory. Findings from this review demonstrate the impacts of and coping strategies to manage uncertainty across the caregiving trajectory, as well as intervention recommendations that build upon caregiver strengths and the *Stroke Caregiver Readiness Model* to help caregivers better reduce and tolerate uncertainty. Subsequent assessments of these recommendations are warranted to determine their effectiveness in promoting caregiver well-being.

## Figures and Tables

**Table 1:** Study demographics.

Included Studies	CG N	SS N	Relation CG N (% Spousal)	Age CG M (SD)	Age SS M (SD)	Gender CG *N* (% Female)	Gender SS *N* (% Female)	Race CG *N* (% White)	Race SS *N* (% White)
Silva-Smith (2007) [24]	12	N/A	7(58%)	58	N/A	9(75%)	N/A	6(50%)	N/A
Lu et al. (2019) [25]	26	N/A	21(81%)	62.7	N/A	20(77%)	N/A	N/A	N/A
Hunt and Smith (2004) [26]	4	4	2(50%)	48.5	58.3	3(75%)	3(75%)	4(100%)	4(100%)
Woodford et al. (2018) [27]	19	N/A	15(79%)	57(15.6)	N/A	17(90%)	N/A	18(94%)	N/A
Brereton and Nolan (2002) [28]	14	N/A	10(71%)	N/A	N/A	8(57%)	N/A	N/A	N/A
Greenwood et al. (2009) [29]	31	N/A	16(52%)	N/A	N/A	22(71%)	17(55%)	22(71%)	N/A
Grant and Davis (1997) [30]	10	10	N/A	48	62	9(90%)	6(60%)	5(50%)	5(50%)
McCurley et al. (2019) [31]	24	N/A	11(46%)	60.5	58.7(16.5)	15(63%)	16(67%)	N/A	N/A
Burman (2001) [32]	13	14	N/A	N/A	N/A	8(61.5%)	N/A	N/A	N/A
Fraser (1999) [33]	1	N/A	0(0%)	N/A	N/A	N/A	N/A	N/A	N/A
O’Connell et al. (2003) [34]	28	N/A	18(64%)	54.9(12.4)	67.6(10.1)	16(57%)	16(57%)	N/A	N/A
Katbamna et al. (2016) [35]	19	N/A	17(89%)	N/A	N/A	15(79%)	8(42%)	12(67%)	12(67%)
White et al. (2014) [36]	15	18	11 (73%)	56.3(14.2)	57.2(8.7)	13(87%)	4(22%)	7(47%)	8(44%)

*Note.* CG = caregiver; SS = stroke survivor; M = mean; SD = standard deviation; N/A = data are not available or not appropriate given study methods.

**Table 2:** Study methods.

Included Studies	Study Aims	Qualitative Method	Sampling Method	Country	Data Collection Location and Timepoints	Interview Length in Minutes Range (M)	Data Analysis Method
Silva-Smith (2007) [24]	To explain the process of preparing for and beginning a new caregiving role	Grounded Theory	Purposive, Consecutive	USA	Semi-structured interviews during inpatient rehabilitation and 4 weeks post-discharge	Not described	Constant Comparative Method
Lu et al. (2019) [25]	To explore the experience of family caregivers in China	Explorative Design	Purposive	China	Semi-structured interviews post-discharge	28–136	Thematic Analysis
Hunt and Smith (2004) [26]	To explore the aftermath of strokes for relatives	Interpretative Phenomenological Analysis (IPA)	Criterion	UK	Semi-structured interviews during inpatient rehabilitation	(45)	IPA
Woodford et al. (2018) [27]	To understand the difficulties experienced by caregivers with anxiety and depression	Not described	Purposive	UK	Semi-structured interviews post-discharge	52–103 (78)	Thematic Analysis
Brereton and Nolan (2002) [28]	To appreciate the needs of new caregivers and how these needs change with role development	Grounded Theory	Purposive	UK	Semi-structured interviews every 2 ± 3 months for up to 18 months during hospital stay and post-discharge	Not described	Constant Comparative Method
Greenwood et al. (2009) [29]	To explore the evolution of caregivers’ experiences over time	Ethnography	Purposive	UK	Open-ended interviews during inpatient rehabilitation and at 1 and 3 months post-discharge	30–90	Not described
Grant and Davis (1997) [30]	To explore losses (of the self) in caregivers	Grounded Theory	Purposive	USA	Semi-structured interviews post-discharge and at a 1 week follow-up	Not described	Constant Comparative Method
McCurley et al. (2019) [31]	To determine challenges and sources of caregiver distress, coping strategies, and ideas for interventions	Elements from Multiple Frameworks	Purposive	USA	Semi-structured interviews during acute hospitalization	20–45	Inductive Qualitative Content Analysis
Burman (2001) [32]	To explore caregiver expectations of the stroke trajectory and management strategies	Grounded Theory	Purposive	USA	Semi-structured interviews post-discharge	30–120	Constant Comparative Analysis
Fraser (1999) [33]	To explore the trajectory of caregiver experiences	Phenomenological	N/A	USA	Eleven unstructured interviews (each 2 weeks apart) during hospitalization and post-discharge	30–60	Time-ordered Matrix
O’Connell et al. (2003) [34]	To determine caregiver perspectives of support and informational needs within hospital and community-based settings	Exploratory Descriptive	Convenience	Australia	Semi-structured interviews during acute hospitalization and post-discharge	20–30	Theme Identification
Katbamna et al. (2016) [35]	To explore factors contributing to stress and strategies to overcome difficulties in White and British Indian caregivers	Ethnography	Purposive	UK	Semi-structured interviews post-discharge (at one and six months)	Not described	Thematic Inductive Framework
White et al. (2014) [36]	To describe the dimensions of uncertainty and strategies to cope with uncertainty for survivors and caregivers	NA	Purposive	USA	Semi-structured focus groups post-discharge	90–130	Content Analysis

*Note.* Post-discharge refers to at-home care.

**Table 3:** Study quality ratings.

Included Studies	Q1 Aims	Q2 Methods	Q3 Design	Q4 Recruitment	Q5 Data Collection	Q6 Researcher Relation	Q7 Ethical	Q8 Data Analysis	Q9 Findings	Q10 Impact
Silva-Smith (2007) [24]	Y	Y	U	Y	Y	N	Y	Y	Y	Y
Lu et al. (2019) [25]	Y	Y	U	Y	Y	Y	Y	Y	Y	Y
Hunt and Smith (2004) [26]	Y	Y	Y	Y	Y	N	Y	Y	Y	Y
Woodford et al. (2018) [27]	Y	Y	U	Y	Y	N	Y	Y	Y	Y
Brereton and Nolan (2002) [28]	Y	Y	U	U	Y	N	N	Y	Y	Y
Greenwood et al. (2009) [29]	Y	Y	U	Y	Y	N	Y	Y	Y	Y
Grant and Davis (1997) [30]	Y	Y	Y	Y	Y	N	N	Y	Y	Y
McCurley et al. (2019) [31]	Y	Y	Y	Y	Y	N	Y	Y	Y	Y
Burman (2001) [32]	Y	Y	U	Y	Y	U	U	Y	Y	Y
Fraser (1999) [33]	Y	Y	Y	N	Y	N	Y	N	U	Y
O’Connell et al. (2003) [34]	Y	Y	U	Y	Y	N	Y	N	U	Y
Katbamna et al. (2016) [35]	Y	Y	Y	Y	Y	Y	Y	Y	Y	Y
White et al. (2014) [36]	Y	Y	U	Y	Y	N	Y	Y	Y	Y

*Note.* Q1–Q10 indicates CASP question number. Coding is as follows: Y = Yes, U = Unsure (cannot be determined by information presented), N = No.

**Table 4:** Lines of argument.

Line of Argument	Third Order Construct	Second Order Construct	First Order Construct	Supporting Reference
Factors Associated with Uncertainty—A Lack of Knowing				
	Cause of the Stroke Event	“They found themselves placed abruptly in an unfamiliar role and experienced a great degree of uncertainty in terms of what had happened to the family member and what having a stroke meant…”	“[I’m] still coming to grips with the situation.”	O’Connell et al. (2003) [34]
	Survivor Prognosis	“A common theme, most pronounced in earlier interviews, was the sense of uncertainty and unpredictability of stroke outcomes that carers had received from clinicians.”	“They just don’t know—that is what is quite scary, it is the unknown. You just don’t know what it is going to be like in a year’s time…because… everybody is just so different.”	Greenwood et al. (2009) [29]
	Safety of Survivor	“They reported feeling uncertain about leaving the stroke survivor alone, through fear of the stroke survivor falling or suffering another stroke, transient ischemic attack (TIA) or post-stroke secondary health complication.”	“It’s just the anxiety that something will happen to him while I’m not there.”	Woodford et al. (2018) [27]
	Availability of Support	“The lack of certainty about the availability of a home care package created additional anxiety about securing the best possible help. Some carers felt that care needs were not adequately assessed, often after numerous calls to social services, but there was also concern about when promised home help would materialize.”	“We were meant to have a care assistant to help with personal care but nothing. It was stressful not knowing if home care services would be provided. For the first few weeks there was no grab rail. His (spouse) left side is quite bad so he could not wash or get dressed himself unaided. They shouldn’t offer services that they couldn’t deliver. It’s very depressing.”	Katbamna et al. (2016) [35]
	Providing Appropriate Care	“…A common expression of feeling ‘left floating by yourself’ was expressed by participants across focus groups.”	“Am I doing things, right? They should have a group there for the family members. To explain what a stroke is. What it does to a person. And what they can do to help them.’’	White et al. (2014) [36]
Impacts of Uncertainty—Concerns about Ability				
	Managing Finances	“Uncertainty about entitlement to cover the costs of essential aids and adaptations meant that carers felt under pressure to pay for them out of their limited resources.”	“When we were getting the essential items, we weren’t adding it all up. … you get a surprise when you see how much it all costs. We thought that the money we were using will be reimbursed but they (Social Services) don’t backdate it. We are very careful how we use our money.”	Katbamna et al. (2016) [35]
	Planning for the Future	“…Once home, continued uncertainty about survivors’ longer term disability and the unreliability of formal support added to difficulties in planning ahead. On a daily basis, survivors’ sometimes unpredictable behavior made forward planning challenging whilst uncertainty about survivors’ abilities made arranging, for example, future holidays hard.”	“I find it (the future) a bit frightening; you know. It is not so much in six months; it is in two or three years’ time, what we are going to be doing? But we can’t plan that far in the future, can we?”	Greenwood et al. (2009) [29]
	Care Options for Survivor	“Additionally, uncertainty around caregivers’ own health in the future was frequently reported, with some caregivers concerned about what would happen to the stroke survivor if they became sick or died themselves.”	“I suppose the way I look at it at the moment is it’s a long dark tunnel and there’s no light at the end of the tunnel at all.”	Woodford et al. (2018) [27]
	Questioning Ability to Cope	“The not knowing seems perpetual. Until there is something more concrete, her uncertainty about her future and her ability to cope will continue. Without definite ideas, participants are left feeling unsettled and all is speculative.”	““How am I going to cope? . . . I don’t know I really don’t know . . . when the worry is on myshoulders that’s all I’m thinking about . . . I don’t think I could take the worry. I don’t—maybe I’ll have to.”	Hunt and Smith (2004) [26]
	Hopelessness	“This [the instability] resulted in an inability to see how things could be improved…”	“…hope is useless…the more you dream, the less you get in reality.”	Lu et al. (2019) [25]
Coping Strategies to Manage Uncertainty				
	Present-focused Thinking	“Despite the diversity of their situations, some coping strategies, for example ‘taking one day at a time’ and ‘living day-by-day’ were mentioned repeatedly. Focusing on the present reduced uncertainty and allowed carers to enjoy everyday things.”	“One day at a time. Take each day and work with the person.”	Greenwood et al. (2009) [29]
	Acceptance	“The caregivers talked about how important it is to accept a new life situation and to not become immobilized by what cannot be changed.”	“If you can do something about it, do it. If you can’t, don’t fuss about it, just accept it and go on to the next thing.”	Burman (2001) [32]
	Gratitude/Positivity	“Carers sometimes described positive outcomes fromthe stroke. Possibly being able to recognize positives allows carers to focus on these rather than the negatives and the uncertainties created by the stroke.”	“It means it does make you appreciate what you have got … and also makes you look at other people and think ‘My God, I am lucky!’ …we are a hell of a lot luckier than some people… We can come and go as we like.”	Greenwood et al. (2009) [29]
	Faith	“Several caregivers used prayer in their safety net.”	“Prayer, that’s the biggest for me, but I admit that I don’t pray as much as I should, but when I start getting out of whack, it’s like … ‘help.’”	Burman (2001) [32]
	Increased Self-Reliance	“The last strategy caregivers used to manage the trajectory was increased self-reliance. Although they sought help from others, they relied on themselves as well.”	“I put that gait belt, or guide belt, on him and get him off of the porch by myself. And I take him around the block by myself. And the other day I walked him 2 blocks by myself and brought him back.”	Burman (2001) [32]
	Information Seeking	“The main form of seeking’ activity here concerned a search for knowledge and understanding. This initial stage was therefore dominated by carers’ attempts to become ‘familiar’ with their situation.”	“Some [nurses] are quite helpful and do come and tell me about my dad and how he has been, others don’t talk to me at all. They know I have read the [medical] notes, but I don’t think I would have been told anything if I hadn’t. I find out most things by reading the notes about his condition. It would be nice if the nurse could find a couple of minutes just to speak to me instead of me having to find the information out for myself.”	Brereton and Nolan (2002) [28]
	Routine Establishment	“Once home, establishing routines was one way of reducing uncertainty. Experienced carers reported their value earlier on whilst newer carers were more likely to describe them later. Routines and planning were seen as better for survivors and carers and gave them greater control over their lives.”	“And we have found–it might be boring, it might be repetitive–but to keep in a routine, you are better off that way.”	Greenwood et al. (2009) [29]
	Duty to Care	“Many caregivers said they felt that it was important to fulfill family obligations as a way to maintain the family structure and patterns.”	“If it was me, I know he would take care of me. And my duty is to take care of him, I think.”	Burman (2001) [32]
	Humor	“Other caregivers became more confident and … saw the humorous side to caregiving.”	“We do joke about his problem with incontinence.”	Katbamna et al. (2016) [35]
	Relational Support	“I find them very good, and I know if I want to go up and say if I need to speak to one of them, they’re willing to, I can ask them anything and they’ll tell me.”	“Support was obtained through the relationship with ward staff, helping participants to cope with adjusting to the changes.”	Hunt and Smith (2004) [26]
	Watchful Monitoring	“Caregivers, especially those who did not live with their loved one, instituted watchful monitoring.”	“It [stroke] put a slightly greater degree of …wanting to be sure I would be over here and just check and make sure everything is okay. To be sure that if anything isn’t okay, I know in the very earliest stages if possible so we can do something when it might actually help.”	Burman (2001) [32]

*Note.* First order = Participant quote from article; Second order = Author interpretation of participant quote from article; Third order = Theme identified in current review.
